# A dataset on spatiotemporal variation of phytoplankton communities in the mangrove wetland of Shenzhen Bay

**DOI:** 10.1038/s41597-025-05357-2

**Published:** 2025-07-01

**Authors:** Zhenfan Chen, Zezhou Zheng, Weicheng Wang, Rao Yao, Yehua Chen, Junjie Li, Shuyan Huang, Kaiqi Xie, Jiangxin Wang, Anping Lei, Haichao Zhou

**Affiliations:** 1 Shenzhen Key Laboratory of Marine Bioresource and Eco-Environmental Science, Shenzhen Engineering Laboratory for Marine Algal Biotechnology, Guangdong Provincial Key Laboratory for Plant Epigenetics, Marine Research Centre, College of Life Sciences and Oceanography, Shenzhen University, Shenzhen, 518060 China; 2 State Key Laboratory of Mariculture Breeding, Key Laboratory of Marine Biotechnology of Fujian Province, Institute of Oceanology, College of Marine Sciences, Fujian Agriculture and Forestry University, Fuzhou, 350002 China; 3 Greater Bay Area Coastal Mangrove Wetland Research & Development Centre, Guangdong Neilingding Futian National Nature Reserve, Shenzhen, 518060 China; 4 Shenzhen Mangrove Wetlands Conservation Foundation, Shenzhen, 518000 China

**Keywords:** Biodiversity, Plant ecology, Marine biology, Biodiversity

## Abstract

The Futian Mangrove Ecological Park (FMEP), a vital subtropical conservation area within Shenzhen Bay, encompasses freshwater lakes and saltwater mangrove wetlands, which have been significantly impacted by human activities over recent decades. Phytoplankton, a key element of the mangrove ecosystem, has experienced over 30 occurrences of harmful algal blooms (HABs) in the coastal waters of Shenzhen Bay since the 1980s. This study presents an extensive dataset documenting the spatiotemporal variation in phytoplankton communities and associated physicochemical parameters within the FMEP. Over a 3-year period from January 2019 to October 2021, 60 water samples were collected at five distinct stations during seasonal surveys, capturing the salinity gradient. Both spatial and temporal variability of phytoplankton community and environmental factors are strongly reflected in our data. Since the dataset was generated using microscopy-based method, it offers precious opportunities for ecological analyses and serves as a valuable reference for biodiversity in future studies, especially in the context of anthropogenic impacts and climate change.

## Background & Summary

Shenzhen Bay, alternatively known as Deep Bay, is a typical subtropical coastal area that has been notably affected by human activities, such as nutrient enrichment and pollution^1–3^. Positioned in a semi-enclosed and shallow setting, the bay is subject to sewage discharge from the burgeoning metropolis of Shenzhen, China. The bay’s entrance, found on the southwest shore, is connected to the Pearl River estuary, which brings in a significant amount of freshwater^4^. The Futian Mangrove Ecological Park (FMEP), situated within the mangrove zones of Shenzhen Bay, is instrumental in supplying organic matter and offering a habitat for aquatic organisms and bird species^5–7^. Nevertheless, in recent decades, eutrophication has become a prominent threat to marine ecosystems^8,9^, with the coastal waters bordering mangrove communities experiencing considerable anthropogenic pollutant influx, thereby endangering the health of mangrove ecosystems^10–12^.

Phytoplankton is a vital component of mangrove ecosystems, with its population dynamics and community structure serving as the initial biological indicators of nutrient enrichment and environmental stressors^13,14^. For instance, in the past nearly 30 years, due to the impact of eutrophication, Shenzhen Bay has experienced over 30 harmful algal blooms (HABs). In addition, the phytoplankton communities near the mangrove zone in Shenzhen Bay have shown long-term variations from 1994 to 2016^15,16^. The bay has witnessed a recurring shift in dominant species, transitioning from dinoflagellates (Dinophyta) and cryptophytes (Cryptophyta) to diatoms (Bacillariophyta), followed by a period dominated by small dinoflagellates. Between 1994 and 2006, small flagellates and *Chroomonas* spp. were the prevalent species in the marine waters. In 2007 and 2009, phytoplankton blooms, including *Skeletonema costatum* and *Thalassiosira* spp., were the primary causative species. In 2010, the community was again primarily composed of small flagellates and *Chroomonas* spp., a trend that continued until 2015. Strikingly, in 2016, there was a significant decline in *Chroomonas* spp., while *Thalassiosira* spp. emerged as the dominant species. These changes in phytoplankton communities and dominant species are likely due to environmental factors and the joint pollution reduction efforts of the Hong Kong and Shenzhen governments^16^. Numerous studies have shown that nutrient enrichment significantly affects phytoplankton at the species and genus levels^17–19^. However, no systematic surveys have been conducted to document the spatiotemporal variations in phytoplankton biodiversity and community structure within the FMEP mangrove ecosystem in recent years.

To enhance our comprehension and strengthen conservation actions for Shenzhen Bay’s mangrove ecosystem, it is crucial to analyse the temporal and spatial dynamics of the phytoplankton community. In the coastal waters adjacent to mangrove regions, harmful algal blooms have intermittently occurred. Phytoplankton growth can be hindered by light limitations due to water mixing and suspended sediments, as well as by ammonium toxicity and predation by zooplankton^4,16^. These coastal zones are consistently subject to intense land-sea interactions. Economic development has led to elevated nutrient levels in most Chinese estuaries and coastal waters, which frequently result in bloom formations^20,21^. Such blooms not only severely impact the mangrove ecosystem, including phytoplankton communities, but also diminish the environmental quality of the FMEP. Consequently, studying the variations in the phytoplankton community in these coastal waters is vital for the surveillance of potential algal blooms. At present, there is a dearth of research on phytoplankton in the coastal waters near FMEP, and the establishment of a comprehensive dataset in this area remains a gap that needs to be addressed.

**Fig. 1:**
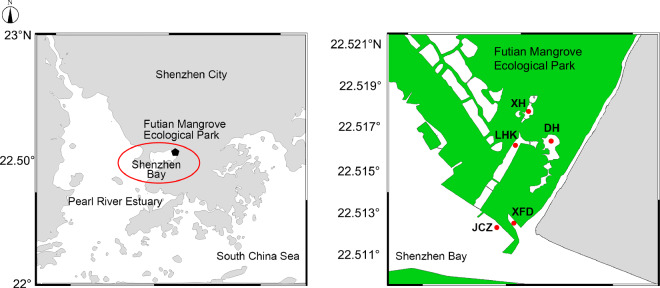
Study area and sampling stations (i.e., XH, DH, LHK, XFD, JCZ) in Futian Mangrove Ecological Park (FMEP), Shenzhen Bay from 2019 to 2021. The green part in the right figure represents the mangrove coverage area.

**Fig. 2:**
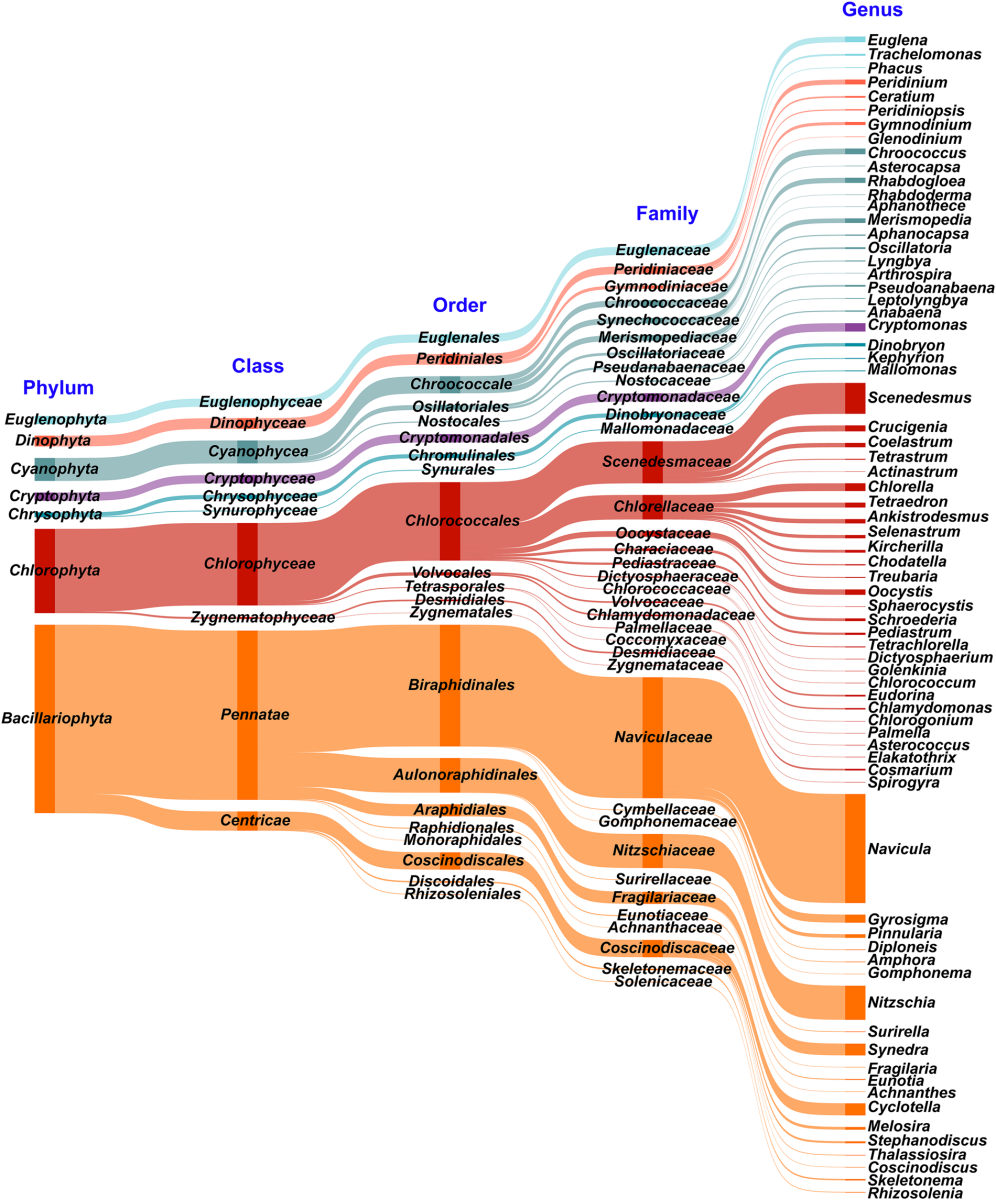
Sankey diagram visualizing the frequency of species in phytoplankton samples collected in FMEP, Shenzhen Bay from 2019 to 2021. The horizontal ordinate of the Sankey diagram at the bottom were taxonomic units at different taxonomy levels. The thickness of each branch presented the frequency of species in their relevant taxonomic units.

**Fig. 3:**
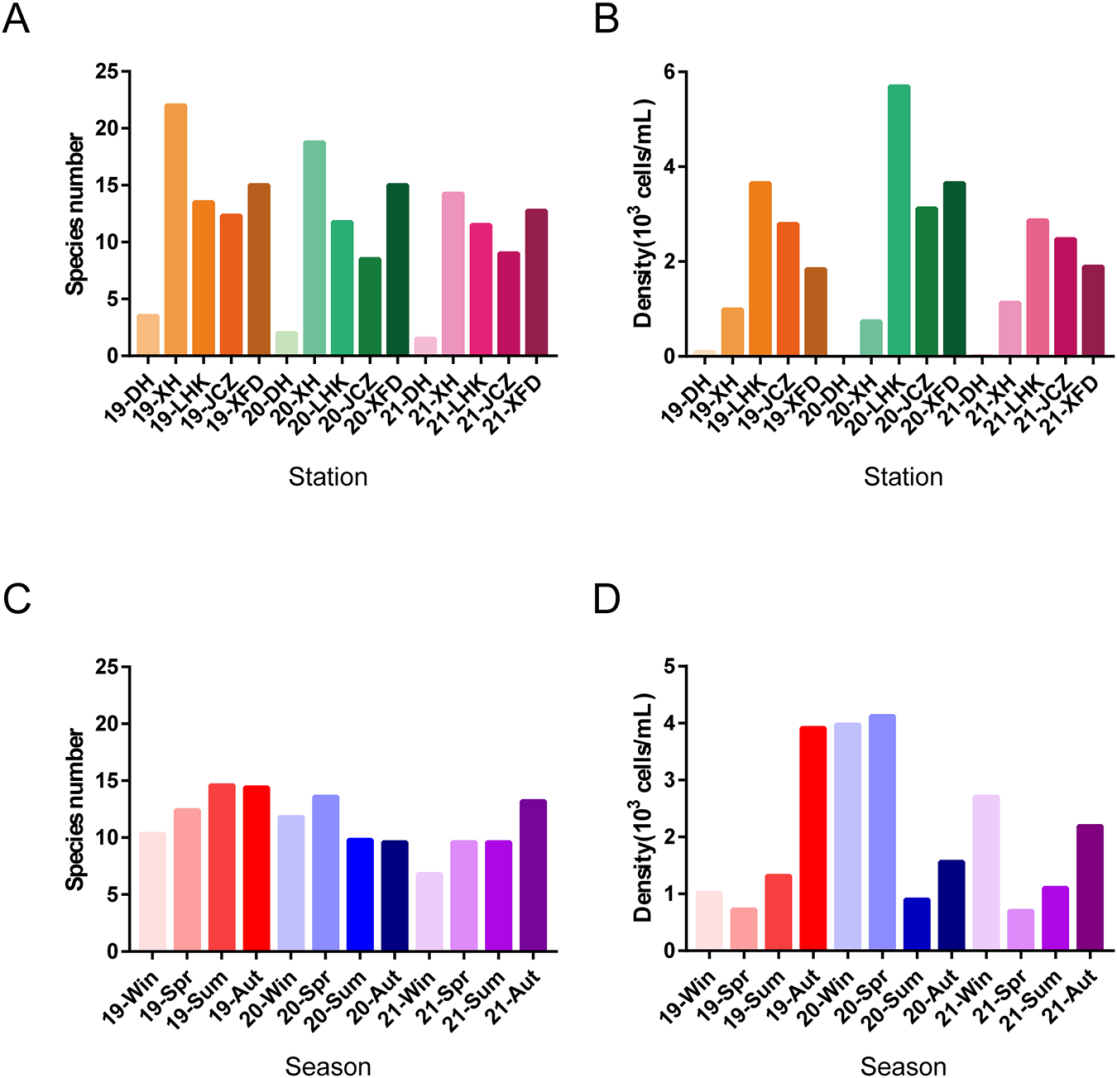
Spatiotemporal dynamics of phytoplankton communities in FMEP, Shenzhen Bay, 2019–2021. (**A**) Average species number across five sampling sites from 2019 to 2021. (**B**) Average cell density of total phytoplankton across five sampling sites from 2019 to 2021. (**C**) Average species number of every season from 2019 to 2021. (**D**) Average cell density of total phytoplankton every season from 2019 to 2021.

**Fig. 4:**
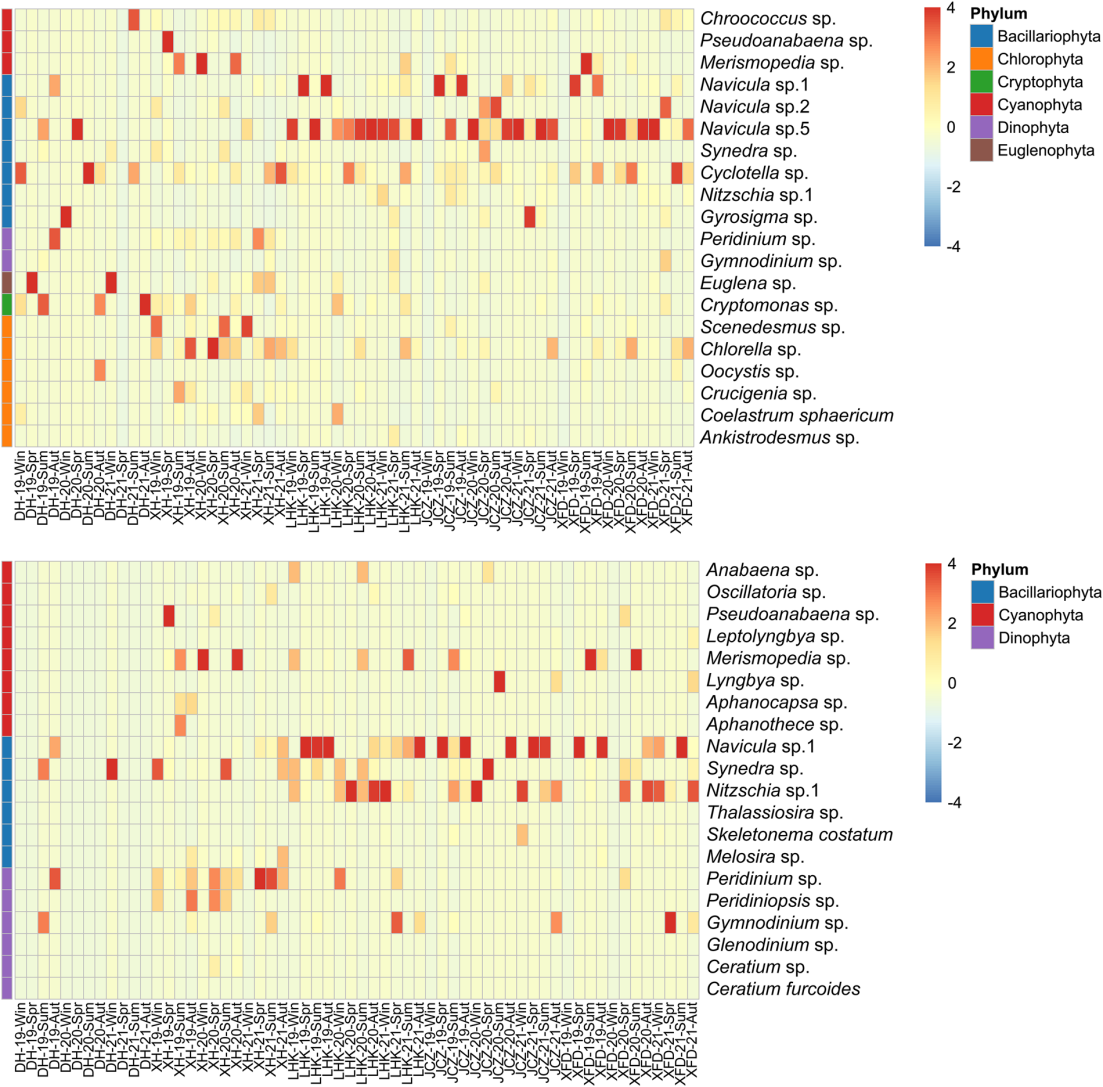
Heatmap analysis of phytoplankton samples from FMEP, Shenzhen Bay (2019–2021). (**A**) Top 20 dominant phytoplankton species based on average abundance, (**B**) Top 20 bloom-forming phytoplankton species based on average abundance. In the heatmap below, the x-axis represents the sample names, while the y-axis on the right indicates different phytoplankton groups. The colour scale reflects deviations from the mean value: negative for values below the mean and positive for values above. The heatmap values are represented by the colour scale in the top-right corner.

In this research, we carried out seasonal sampling of phytoplankton and environmental factors from January 2019 to October 2021 in Shenzhen Bay, near the FMEP, located south of Futian District, Shenzhen, China (Fig. 1). Throughout the three-year study, we identified a total of 72 genera and 89 taxa of phytoplankton within the FMEP, spanning across 7 phyla: Bacillariophyta, Chlorophyta, Chrysophyta, Cryptophyta, Cyanophyta, Dinophyta, and Euglenophyta (Fig. 2, Table 1). Chlorophyta was the most diverse, comprising 28 genera and 34 taxa; Bacillariophyta followed with 19 genera and 28 taxa. Cyanobacteria constituted the third largest group, with 13 genera and 13 taxa. Other algal species were less abundant, summing up to 12 genera and 14 taxa. Spatially, the species number and cell density of phytoplankton exhibited consistent patterns across our sampling stations over the three-year period (Fig. 3A, B). However, the seasonal variations in species number and cell density of phytoplankton communities showed differences between years (Fig. 3C,D). We detected numerous potential bloom-forming species or harmful algal species, including *Merismopedia* and *Pseudoanabaena* in Cyanophyta; *Navicula*, *Synedra*, *Cyclotella*, *Thalassiosira*, *Skeletonema*, *Pinnularia*, and *Nitzschia* in Bacillariophyta; *Peridiniopsis* and *Gymnodinium* in Dinophyta; and *Cryptomonas* in Cryptophyta, among others (Fig. 4).

**Table 1:** Frequency of observations distributed among taxonomic phytoplankton phyla and classes present in phytoplankton samples collected in FMEP, Shenzhen Bay from 2019 to 2021.

Taxonomic Class	Number of observations	Percentage of observations
Chlorophyceae	207	31.46%
Pennatae	176	26.75%
Cyanophycea	86	13.07%
Centricae	72	10.94%
Dinophyceae	37	5.62%
Cryptophyceae	31	4.71%
Euglenophyceae	30	4.56%
Chrysophyceae	9	1.37%
Zygnematophyceae	7	1.06%
Synurophyceae	3	0.46%

This comprehensive, multi-year monitoring dataset offers a wealth of valuable insights for studying the dynamics of phytoplankton community succession and the potential bloom-forming species or harmful algal species in the mangrove regions of Shenzhen Bay. It is an invaluable asset for assessing the impact of human activities, especially pollution, on the health and stability of these critical mangrove ecosystems.

## Methods

### Sampling

In our study, we established five sampling sites near the Futian Mangrove Ecological Park (FMEP) within Shenzhen Bay. Nestled against the western boundary, adjacent to the Futian National Nature Reserve and separated from the Hong Kong Mai Po Nature Reserve, this area, encompassing about 38 hectares, is a vital part of the Shenzhen Bay Wetland ecosystem. The sampling sites, labelled as XH (22.514°N, 114.035°E), DH (22.513°N, 114.036°E), LHK (22.511°N, 114.033°E), JCZ (22.507°N, 114.032°E), and XFD (22.509°N, 114.034°E), are illustrated in Fig. 1. XH and DH, being freshwater inner lakes, are in stark contrast to JCZ and XFD, which are situated at the periphery of the mangroves, while LHK acts as the nexus between the inner lakes and Shenzhen Bay. This placement of sites allows for a comprehensive examination of phytoplankton community dynamics influenced by both freshwater and seawater in the FMEP area. From January 2019 to October 2021, we conducted seasonal (i.e. January, April/May, July, October) water sampling at these five locations. The sampling protocol included: (1) The collection of a 2-liter surface water sample using a plexiglass water collector from BJT, China; (2) The preservation of samples with a 15‰ solution of Lugol’s reagent for 24 to 36 hours, followed by concentration from 2 liters to 150 ml; (3) The transfer of the concentrated samples to a quantitative sample bottle for the identification of phytoplankton.

### Measurement of physical and chemical parameters

At each sampling site, the physicochemical parameters of the waters, including water temperature (°C), salinity, dissolved oxygen (DO, mg/L), total nitrogen (TN, g/Kg), and total phosphorus (TP, g/Kg), were determined in accordance with the specification for marine monitoring-Part 4: Seawater analysis by State Oceanic Administration of the People’s Republic of China (2007)^22^. Specifically, surface water samples (1 L each) were collected using a water sampler and stored in plastic bottles for physicochemical analysis. Water temperature, salinity, and DO were measured *in situ* utilizing a multiparameter water quality meter (Multi 3630, WTW, Germany). All samples were promptly transported to the laboratory in a cooler and preserved at 4 °C. For nutrient determination, the water samples were immediately filtered through 0.45 μm pore glass fiber filters (GF/F, Whatman). The TN concentration was measured using the alkaline potassium persulfate oxidation method with an accuracy of 0.01 mg N/L, while the TP concentration was determined by phosphorus molybdenum blue spectrophotometry with an accuracy of 0.005 mg/L.

### Phytoplankton identification

The samples were observed under DMi3000B inverted microscope (Leica, Germany) with a magnification of 400×. After shaking the sample well, 100 μL of concentrated sample water was transferred to the phytoplankton counting frame, then the algal species were identified by morphology and counted. At last, the qualitative and quantitative results of phytoplankton were obtained for further analysis, the numbers of the phytoplankton in the samples were counted and standardized into density (cells/mL).

### Measurement of phytoplankton biodiversity

The species diversity index can be used to measure both the number of species and the uniformity of their distribution^23^. In this study, the Shannon-Weiner diversity index (*H*′) (1), Margalef species richness index (*d*) (2) and Simpson’s index (*D*) (3) were calculated.

1 $${H}^{{\prime} }\,=\,\sum ({Pi})(\mathrm{ln}{Pi})$$

2 $$d=(S-1)/\mathrm{ln}N$$

3 $$D=1-\sum (Pi\wedge 2)$$

where $\,{Pi}={ni}/N$, *ni* represents the number of individuals of the i-th species in the sample, *N* represents the total number of individuals of all species in the sample, and S represents the number of species of all species in the sample.

## Data Records

The dataset is available at figshare repository^24^. The data contains three files. The first file, “Shenzhen Bay Phytoplankton Abundance Data.xlsx” contains the phytoplankton abundance data set, which includes 60 samples collected from 5 stations in Futian Mangrove Ecological Park, Shenzhen Bay. The sampling was conducted quarterly, and continuously sampled from 2019 to 2021. The data contain 60 rows (samples) and 100 columns including 3 time parameters (year, season and month), 3 spatial parameters (station, latitude and longitude), 4 phytoplankton biodiversity index (species number, Shannon-Weiner diversity index, Margalef species richness index, Simpson’s index), and 90 phytoplankton abundances (total phytoplankton abundance and 89 species abundances in 10^3^ ind./mL). The second file, “Shenzhen Bay Environmental Variables Data.xlsx” is a surface seawater environmental data measured by an independent party at the same sampling stations and time periods. This file includes 3 time parameters (year, season and month), 3 spatial variables (station, latitude and longitude), 6 environmental factors [water temperature (°C), salinity, dissolved oxygen (DO, mg/L), total nitrogen (TN, g/kg), total phosphorus (TP, g/kg), and TN/TP]. The third file, “Sankey diagram and Heatmap.r”, is the R-code used to produce Figs. 2 and 4.

## Technical Validation

To ensure the accuracy of the data and metadata, rigorous data curation and technical validation steps were implemented. During data collection, a standardized sampling protocol, previously used in studies^4,25^, was followed to avoid bias and ensure the replicability of the data and information. All samples were collected, identified, and measured by a team of qualified researchers and taxonomists. Data quality was maintained through careful validation of taxonomic and morphological classifications of phytoplankton, as well as detection and correction of format and nomenclature errors, missing data, and inconsistencies. The dataset underwent thorough taxonomic and morphological verification using the World Register of Marine Species (WoRMS) database (http://www.marinespecies.org/). Associated abiotic data is available for most samples, though some exceptions exist. The dataset allows for analyses of spatial and temporal variations in phytoplankton community structure and has already been published in a peer-reviewed scientific journal^25^.

## Data Availability

Code for phytoplankton data processing and for reproducing the graphs of this paper is available through our figshare repository^24^.
